# Ventriculo‐arterial coupling detects occult RV dysfunction in chronic thromboembolic pulmonary vascular disease

**DOI:** 10.14814/phy2.13227

**Published:** 2017-04-03

**Authors:** Richard G. Axell, Simon J. Messer, Paul A. White, Colm McCabe, Andrew Priest, Thaleia Statopoulou, Maja Drozdzynska, Jamie Viscasillas, Elizabeth C. Hinchy, James Hampton‐Till, Hatim I. Alibhai, Nicholas Morrell, Joanna Pepke‐Zaba, Stephen R. Large, Stephen P. Hoole

**Affiliations:** ^1^ Medical Physics and Clinical EngineeringCambridge University Hospital NHS Foundation TrustCambridgeUK; ^2^ Postgraduate Medical InstituteAnglia Ruskin UniversityChelmsfordUK; ^3^ Department of Cardiovascular SurgeryPapworth Hospital NHS Foundation TrustCambridgeUK; ^4^ Pulmonary Vascular Diseases UnitPapworth Hospital NHS Foundation TrustCambridgeUK; ^5^ The Royal Veterinary CollegeHatfieldLondonUK; ^6^ MRC Mitochondrial Biology UnitCambridge Biomedical CampusCambridgeUK; ^7^ Department of Interventional CardiologyPapworth Hospital NHS Foundation TrustCambridgeUK

**Keywords:** Chronic thromboembolic disease, chronic thromboembolic pulmonary hypertension, right ventricular dysfunction, ventriculo‐arterial coupling

## Abstract

Chronic thromboembolic disease (CTED) is suboptimally defined by a mean pulmonary artery pressure (mPAP) <25 mmHg at rest in patients that remain symptomatic from chronic pulmonary artery thrombi. To improve identification of right ventricular (RV) pathology in patients with thromboembolic obstruction, we hypothesized that the RV ventriculo‐arterial (Ees/Ea) coupling ratio at maximal stroke work (Ees/Ea_max sw_) derived from an animal model of pulmonary obstruction may be used to identify occult RV dysfunction (low Ees/Ea) or residual RV energetic reserve (high Ees/Ea). Eighteen open chested pigs had conductance catheter RV pressure‐volume (PV)‐loops recorded during PA snare to determine Ees/Ea_max sw_. This was then applied to 10 patients with chronic thromboembolic pulmonary hypertension (CTEPH) and ten patients with CTED, also assessed by RV conductance catheter and cardiopulmonary exercise testing. All patients were then restratified by Ees/Ea. The animal model determined an Ees/Ea_max sw_ = 0.68 ± 0.23 threshold, either side of which cardiac output and RV stroke work fell. Two patients with CTED were identified with an Ees/Ea well below 0.68 suggesting occult RV dysfunction whilst three patients with CTEPH demonstrated Ees/Ea ≥ 0.68 suggesting residual RV energetic reserve. Ees/Ea > 0.68 and Ees/Ea < 0.68 subgroups demonstrated constant RV stroke work but lower stroke volume (87.7 ± 22.1 vs. 60.1 ± 16.3 mL respectively, *P* = 0.006) and higher end‐systolic pressure (36.7 ± 11.6 vs. 68.1 ± 16.7 mmHg respectively, *P* < 0.001). Lower Ees/Ea in CTED also correlated with reduced exercise ventilatory efficiency. Low Ees/Ea aligns with features of RV maladaptation in CTED both at rest and on exercise. Characterization of Ees/Ea in CTED may allow for better identification of occult RV dysfunction.

## Introduction

Chronic thromboembolic pulmonary hypertension (CTEPH) occurs when organized thrombi obstruct the pulmonary arteries (PA) increasing pulmonary vascular resistance (PVR) and in turn elevating the mean PA pressure (mPAP) (Simonneau et al. 2013). There is a subgroup of patients who present with chronic thromboembolic disease (CTED), who have mPAP < 25 mmHg at rest, but who are nevertheless symptomatic and possibly deserving of treatment. Right Ventricular (RV) dysfunction in these patients may limit pulmonary pressure generation both at rest and on exercise resulting in underestimation of disease severity. Invasive hemodynamic assessment by right heart catheterization is the gold standard diagnostic test and these data are the primary determinant of prognosis (Champion et al. 2009). However, it is pressure focused and patients with CTED and occult RV dysfunction that is contributory to their symptoms can be overlooked (Castelain et al. 2001; Gan et al. 2007). The under‐diagnosis of RV dysfunction in CTED and the limited understanding of its clinical course are significant unmet clinical needs. The concept of ventriculo‐arterial (Ees/Ea) coupling and RV energetic reserve may offer novel insight in to the pathophysiology of CTED and CTEPH and more accurately define the disease state.

Pressure‐volume (PV)‐loop methodology allows for RV‐PA Ees/Ea coupling to be defined as the matching between RV contractility (End‐systolic elastance – Ees) and PA afterload (Effective arterial elastance – Ea) (Kelly et al. 1992). There has been an emerging trend of clinical studies performed using Ees/Ea coupling to detect changes in patients with different PH etiologies and disease severity. In patients with a clinical diagnosis of CTED/CTEPH (McCabe et al. 2014), PH and associated systemic sclerosis (Tedford et al. 2013), and those with PH and without overt RV failure (Kuehne et al. 2004), it was demonstrated that Ees/Ea uncoupling was due to a disproportional increase in Ea and the inability to augment contractility (Ees). These observations suggest that in early stage PH, decreased Ees/Ea coupling is mainly reflected in increased afterload (Ea) and insufficient augmentation of RV contractility. However, in late stage PH, the continued progression of the disease state leads to the clinical development of RV failure and further impairs Ees/Ea coupling due to decreased contractility (Ees) (Kuehne et al. 2004). While there is potential for Ees/Ea coupling to be used as a marker of RV function and illustrate the transition from RV adaptation to RV maladaptation, it still remains unclear at what specific coupling ratio the RV becomes uncoupled to the PA. Previous animal model data suggests maximal stroke work (SW) was achieved at an Ees/Ea_max sw_ = 0.6 mmHg/mL, and RV dysfunction ensued with any further increase in afterload. However, this study determined Ees from a PA occlusion that is now known to overestimate true Ees, and Ea was determined from mean RV pressures rather than the end‐systolic pressure (ESP).

In health, the RV‐PA is coupled to maintain optimal cardiac output but it is possible to determine the maximal SW in response to an acute artificial increase in afterload. The Ees/Ea coupling ratio at which this point is reached is termed Ees/Ea_max sw_. Above this threshold there is RV energetic reserve, however, if this threshold is not maintained there is no energetic reserve and RV dysfunction will ensue (Burkhoff and Sagawa 1986). We hypothesized that Ees/Ea_max sw_ defined in an animal model may be applied clinically to detect occult RV dysfunction in patients with CTED and potentially identify those patients with CTEPH who retain RV energetic reserve. To corroborate the presence or absence of RV energetic reserve, contemporaneous cardiopulmonary exercise test data, where available, was also analyzed in patients.

## Methods

### Animal model

Eighteen open‐chested large white swine with a median weight of 77 kg (range 67–92 kg) were studied.

#### Anesthetic protocol and lines

Swine were fasted for 12 h leading up to the procedure. Water was allowed until one hour before premedication. Premedication was undertaken with intramuscular injection of midazolam (0.5 mg/kg), ketamine (15 mg/kg) and atropine (0.02 mg/kg). Induction of anesthesia was performed using intravenous (IV) injection of fentanyl (5.0 mcg/kg) over 5 min and propofol (2.0–4.0 mg/kg) administered to effect. Oral endotracheal intubation was established for institution of mechanical ventilation with a respiratory rate of 12–15 breaths per min and tidal volume 10 ml/kg. The ventilator was set with volume control with peak pressure <12 cmH_2_O and positive end‐expiratory pressure of 5 cmH_2_O. Paralysis was induced with an IV injection of pancuronium (0.15 mg/kg). Anaesthesia was maintained using a constant infusion of fentanyl (0.2 mcg/kg/min) and propofol (0.2 mg/kg/min). Arterial and venous cannulas were located for measurement of arterial blood pressure and central venous pressure, respectively, using multi‐parameter anesthesia monitor. Prior to placing the conductance catheter into the RV an IV injection of lidocaine (100 mg, total dose) was administered.

#### Catheterization

**Figure 1:**
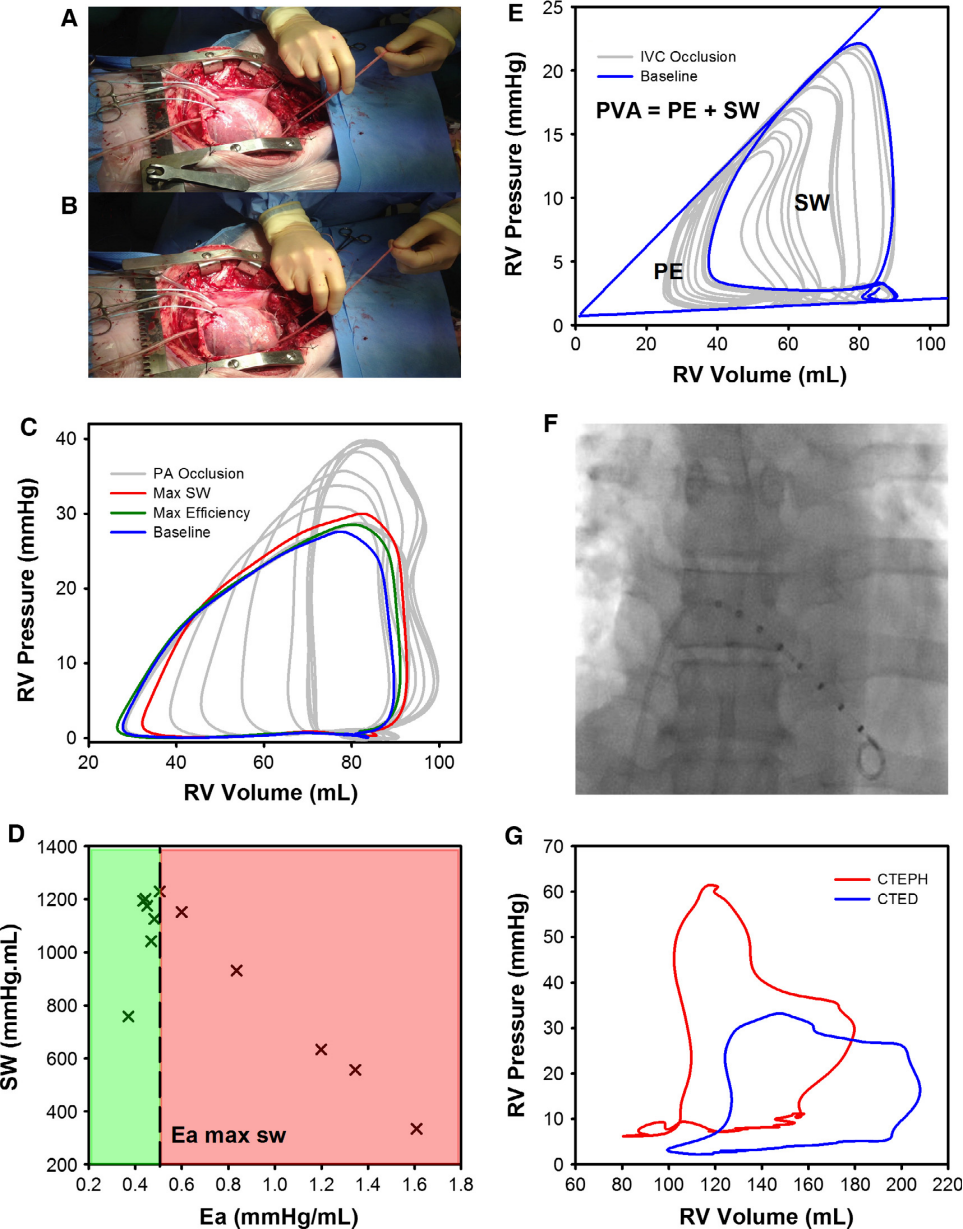
(A) Animal model porcine heart instrumented with conductance catheter; (B) with PA snare partially occluded to increase RV afterload; (C) RV PV‐loops recorded during the PA snare. PV‐loops are highlighted at baseline (blue), maximal efficiency (green) and maximal SW (Red). (D) SW‐Ea RV PV‐loop relationship during the PA snare, (green) RV energetic reserve, (red) RV failure. (E) RV PV‐loops recorded during an IVC occlusion. PV‐loops are highlighted at baseline (blue). SW is highlighted as the area contained within the baseline RV PV‐loop. PE is the area within the Ees and EDPVR pressure volume relationships. (F) Fluoroscopic image of conductance catheter located in the RV during the clinical study; (G) Typical RV PV‐loop morphology recorded for patients with CTED (blue) or CTEPH (red).

Routine Swan‐Gantz catheterization was performed via a 7‐F sheath placed in the right jugular vein. Room temperature saline injected in triplicate was used to determine cardiac output by the standard thermodilution technique (CO_TD_). A midline sternotomy was performed and the pericardium was removed. A 7‐F, 8 electrode conductance catheter (Millar Instruments, Houston, TX) was inserted through a puncher in the RV apex and placed along the long axis of the ventricle (Fig. 1). PV‐loop morphology was used to confirm the correct placement of the catheter within the RV.

### Clinical study

Ten patients with a clinical diagnosis of CTEPH and 10 patients with a clinical diagnosis of CTED were invited to participate. Exclusion criteria were a myocardial infarction in the preceding 12 weeks, previous permanent pacemaker implant or atrial fibrillation.

#### Catheterization

Routine Swan‐Ganz catheterization was performed via a 7‐F sheath placed in the right femoral or jugular vein using lidocaine local anesthetic. The catheter was advanced and positioned in the right atrium (right atrial pressures were measured), the RV and finally the PA (PA pressures were measured). Cardiac output was determined by thermodilution (CO_TD_) in triplicate using 10 mL cool saline injection. A 7‐F, eight electrode conductance catheter was then inserted through the venous sheath, advanced across the tricuspid valve and placed along the long axis of the ventricle in to the RV apex under fluoroscopic guidance (Fig. 1).

### Cardiopulmonary exercise testing

Where able, patients underwent symptom‐limited incremental cardiopulmonary exercise testing contemporaneous to right heart and conductance catheterization procedures (within 1 week). Exercise testing was undertaken according to ATS protocols aiming for an optimal exercise test duration of 8–12 min.

### Conductance calibration

A 20‐kHz 30 μA current was applied to the outer most electrodes to generate an intra‐cavity electric field. The time varying conductance, *G(t)*, was calculated by measuring the sum of the conductance between the five remaining sensing electrode pairs. The conductance catheter was calibrated for parallel conductance *G(p)* using the hypertonic saline technique first described in the LV by Baan et al. (1984) and has subsequently been used for the RV (McKay et al. 1984; McCabe et al. 2014). The time varying volume, *V(t)*, was calculated as follows: *V*(*t*) = 1/*α* × *L* ^2^/*σx*[*G*(*t*) ‐ *G*(*p*)]; *α* the ratio of the conductance‐derived volume to the true volume calculated by CO_TD_), *L* is the inter‐electrode distance, *σ* is the blood conductivity (the reciprocal of the specific resistivity of the blood measured directly with the Millar cuvette) and *G(p)* is the parallel conductance (conductance of fluids and tissues surrounding the RV).

### Pressure volume loop data acquisition

#### Animal model

The conductance technique was used to measure the PV‐loop relationship during ventilation suspension to provide a beat‐to‐beat assessment of RV function at steady state for at least 5‐cardiac cycles (Fig. 1). Two families of PV‐loops were recorded during ventilation suspension. A snare was looped around the inferior vena cava (IVC) and partially occluded to record a family of PV‐loops during a preload reduction. A separate snare was looped around the PA and partially occluded over a 10–15 sec period so that multiple data points could be recorded to determine how RV hemodynamic parameters varied with acute changes in PA afterload (Fig. 1).

#### Clinical study

The conductance technique was used to measure the PV‐loop relationship during ventilation breath hold at end‐expiration to provide a beat‐to‐beat assessment of RV function at steady state for at least 5‐cardiac cycles (Fig. 1). It was not feasible to perform an IVC occlusion in the clinical study.

### Off‐line RV hemodynamic measurements

The conductance catheter data was analyzed offline using LabChart software (LabChart 7.0, ADInstruments, NSW, Australia). Five steady‐state PV‐loops were recorded to generate load‐dependent parameters of systolic and diastolic function. The systolic parameters of ventricular function were stroke work (SW), cardiac output (CO), stroke volume (SV), ejection fraction (EF), end‐systolic pressure (ESP) and the maximum rate of isovolumic contraction (dP/dt_max_). The diastolic parameters of ventricular function were end‐diastolic pressure (EDP), the maximum rate of isovolumic relaxation (dP/dt_min_), the effective arterial elastance (Ea) (Kelly et al. 1992) and the time constant of diastolic relaxation (Tau) (Weiss et al. 1976; Raff and Glantz 1981; Matsubara et al. 1995). Ea is defined as the ratio of end‐systolic pressure (ESP) to stroke volume (SV) and characterizes PA afterload from a combination of PVR, pulmonary artery compliance and inductance (Sunagawa et al. 1983, 1985; Morimont et al. 2008), to provide a considerably better estimate of system behaviour than PVR alone (Greyson 2010). Tau represents the exponential decay of the RV pressure during isovolumic relaxation. Although Tau is considered load‐dependent, it is predominantly affected by heart rate, with an increased heart rate reducing the time for diastolic filling. At a steady heart rate it can be considered load‐independent.

#### Animal model

A family of PV‐loops recorded during an IVC snare were used to determine the Ees. Ees is the gradient of the slope of the end systolic pressure volume relationship (ESPVR) and is a load‐independent parameter of systolic contractility. Ees can be used with Ea to determine the ventriculoarterial‐coupling ratio (Ees/Ea). A family of PV‐loops was recorded during a PA snare to determine how systolic and diastolic parameters of RV function (as described above) vary in response to increasing afterload (Ea) and ventriculoarterial‐coupling (Ees/Ea).

PV‐loop morphology was used to determine maximal RV efficiency from the SW/pressure volume area (PVA) relationship. Ventricular efficiency is defined as the ratio between SW and oxygen consumption (Burkhoff and Sagawa 1986). PVA is a linear surrogate of myocardial oxygen consumption (Suga et al. 1980, 1983, 1984). PVA is the total mechanical work performed by the ventricle, calculated from the sum of the external stroke work (SW – determined from the area contained within the PV‐loop) that propels the blood from the ventricle and mechanical potential energy (PE – the area bound by the ESPVR and EDPVR) stored in the ventricle at the end of each contraction (Fig. 1) (Suga 1979).

#### Clinical study

Single beat estimation of Ees was performed using a Matlab (MathWorks, MA) sinusoidal curve‐fitting algorithm to estimate the theoretical maximal isovolumic pressure (*P*_max_) from a nonlinear extrapolation of the dP/dt_max_ and dP/dt_min_ points on the RV pressure waveform (Brimioulle et al. 2003). Ees was then calculated as the slope of the *P*_max_‐ESP divided by the SV (Brimioulle et al. 2003).

### Statistical analysis

Data are expressed as mean ± standard deviation and *P*‐values were calculated using a two‐tailed unpaired student t test for pairwise comparisons, and a Kruskal–Wallis one‐way ANOVA on Ranks followed by Tukey's test for multiple comparisons, unless otherwise stated. Categorical data are expressed as number (percentage) and compared with the Fisher Exact test. Linear regression analysis was used to determine Ees. A *P* < 0.05 was considered statistically significant. Analysis was performed using SigmaPlot 12.5 (Systat Software Inc, San Jose CA) statistical analysis package. The primary outcome measure was Tau. The number of subjects required was informed from previous work (Read et al. 2011). To detect a change in Tau of 10 ± 8 msec between groups, we estimated 11 patients would be required (*α* = 0.05, *β* = 0.2). We decided to recruit 20 to account for possibly incomplete or poor quality datasets.

### Ethics

#### Animal model

The animal study was approved by the Home Office (Project Licence 70/7967). All animals received humane care and the animal study adhered to the Code of practice for the housing and care of animals bred, supplied, or used for scientific purposes (Animals (Scientific) Procedures Act, 1986, published 2014). The animal study was performed to conform to the guidelines from Directive 2010/63/EU of the European Parliament on the protection of animals used for scientific purposes.

#### Clinical study

The clinical study was approved by the local research ethics committee (REC number 12/EE/0085), and complied with the guidelines set out in the Declaration of Helsinki. All participants gave written informed consent.

## Results

### Animal model

**Figure 2:**
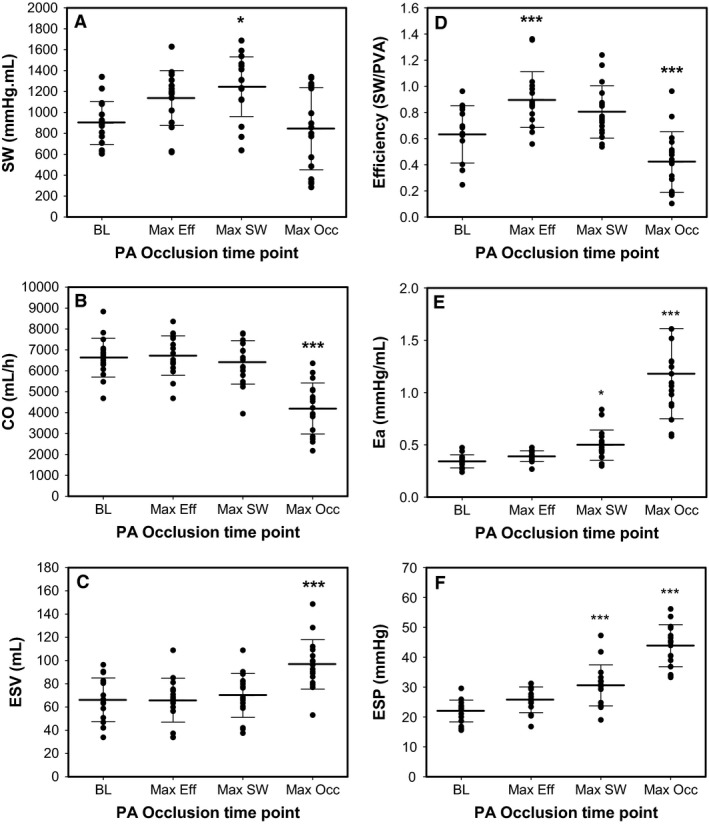
Animal model (*n* = 18) RV hemodynamic data for (A) SW; (B) CO; (C) ESV; (D) SW/PVA; (E) Ea; and (F) ESP at baseline (BL) and the PA occlusion time point for maximal efficiency (Max Eff), maximal SW (Max SW) and maximal PA occlusion (Max Occ.). *P < 0.05; **P < 0.01; ***P < 0.001.

**Figure 3:**
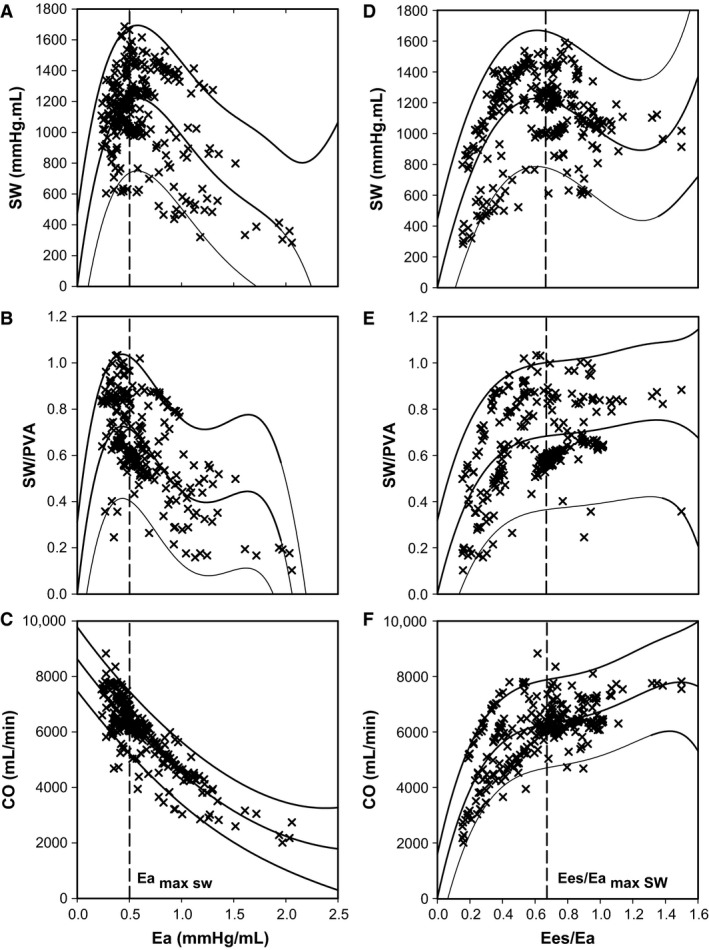
Animal model relation between afterload quantified by Ea and (A) SW; (B) SW/PVA; and (C) CO. Animal model relation between the ventriculo‐arterial coupling ratio (Ees/Ea) and (D) SW; (E) SW/PVA; and (F) CO. Pooled data from 18 hearts are shown (*n* = 324). Mean regression lines with 95% prediction intervals are displayed.

Baseline hemodynamic data are shown in Table 1. The porcine model had similar hemodynamic data and weight to humans. At baseline, none of the pigs had pulmonary hypertension or evidence of RV dysfunction. Conductance catheter data at baseline and then during the PA snare are described in Table 2 and Fig. 2. This shows the RV was modestly uncoupled at baseline (0.94 ± 0.18) and maximal efficiency occurred at a higher Ees/Ea than maximal SW: Ees/Ea 0.84 ± 0.23 versus 0.68 ± 0.23, respectively. In terms of RV afterload, maximal efficiency was achieved at a lower Ea than maximal SW: Ea (mmHg/mL) 0.39 ± 0.05 versus 0.50 ± 0.14, respectively (Fig. 3). While HR (*P* = 0.829) and Tau (*P* = 0.930) remained constant there was a significant increase in dP/dt_min_ (*P* < 0.001), explained by the load‐dependency of dP/dt_min_ and the significant rise in ESP (*P* < 0.001) whilst EDP remained constant (*P* = 0.626). Below the Ees/Ea_max sw_ coupling ratio threshold of 0.68, RV SW, efficiency, and CO declined.

**Table 1:** Animal model hemodynamic data

	Group (*n *=* *18)
Data	
Weight, kg	78.8 ± 6.7
Height, cm	128.2 ± 5.2
Heart rate, BPM	101.3 ± 2.0
MAP, mmHg	78.5 ± 12.6
MPAP, mmHg	20.3 ± 3.4
SvO_2_, %	65.5 ± 8.7
CO_TD_, L/min	6.6 ± 1.1
CI_TD_, L/min/m^2^	4.2 ± 0.6

Values are mean ± SD.

MAP, mean arterial pressure; MPAP, mean pulmonary arterial pressure; SvO_2_, mixed venous oxygen saturation; CO, cardiac output; CI, cardiac index; TD, thermodilution.

**Table 2:** Animal model RV hemodynamic data at baseline, maximal efficiency, maximal SW and during the maximal PA occlusion

	Baseline	Maximal efficiency	Maximal SW	Maximal PA occlusion	*P*‐value
CO, L/min	6.6 ± 0.9	6.7 ± 0.9	6.4 ± 1.1	4.2 ± 1.2	<0.001
ESV, mL	66.1 ± 18.8	65.9 ± 18.9	70.0 ± 18.9	96.7 ± 21.3	<0.001
EDV, mL	107.7 ± 18.5	105.6 ± 18.5	106 ± 19.4	120.5 ± 22.3	0.103
SV, mL	65.2 ± 8.9	66.1 ± 8.9	63.1 ± 9.8	40.7 ± 11.6	<0.001
EF, %	64.0 ± 6.6	65.5 ± 9.0	61.3 ± 8.8	35.4 ± 9.9	<0.001
ESP, mmHg	22.0 ± 3.7	25.7 ± 4.3	30.6 ± 6.9	43.9 ± 7.0	<0.001
EDP, mmHg	5.2 ± 3.0	5.8 ± 3.2	5.7 ± 3.0	6.6 ± 3.2	0.626
HR, BPM	101.7 ± 1.8	101.8 ± 1.8	101.4 ± 1.8	103.2 ± 6.1	0.829
dp/dt_max_, mmHg/sec	307 ± 61	352 ± 77	350 ± 71	344 ± 55	0.176
dp/dt_min_, mmHg/sec	−217 ± 36	−248 ± 50	−299 ± 56	−360 ± 54	<0.001
Tau, msec	61.5 ± 27.0	58.7 ± 27.4	56.6 ± 25.0	61.5 ± 23.6	0.930
Ea, mmHg/mL	0.34 ± 0.06	0.39 ± 0.05	0.50 ± 0.14	1.18 ± 0.43	<0.001
SW, mmHg.mL	899 ± 205	1138 ± 263	1246 ± 285	844 ± 393	0.002
PVA, mmHg.mL	1616 ± 709	1327 ± 424	1614 ± 483	2152 ± 621	0.002
SW/PVA	0.63 ± 0.22	0.90 ± 0.21	0.80 ± 0.20	0.42 ± 0.23	<0.001
Ees/Ea	0.94 ± 0.18	0.84 ± 0.23	0.68 ± 0.23	0.32 ± 0.15	<0.001

Values are mean ± SD.

CO, cardiac output; ESV, end‐systolic volume; EDV, end‐diastolic volume; SV, stroke volume; EF, ejection fraction; ESP, end‐systolic pressure; EDP, end‐diastolic pressure; HR, heart rate; dP/dt max, maximum rate of isovolumic contraction; dP/dt min, maximum rate of isovolumic relaxation; Tau, time constant of diastolic relaxation; Ea, effective arterial elastance; SW, stroke work; PVA, pressure volume area; SW/PVA, RV efficiency; Ees/Ea, ventriculoarterial coupling ratio.

### Clinical study

Demographic human data from 10 patients with CTEPH and 10 patients with CTED are shown in Table 3. We confirmed that the mPAP (*P* < 0.001) and PVR (*P* < 0.001) were normal in the CTED group and elevated in the CTEPH group. Ea (*P* = 0.005) was elevated in the CTEPH group. While HR (*P* = 0.560) and Tau (*P* = 0.954) remained constant, there was a significant increase in dP/dt_max_ (*P* = 0.016) and dP/dt_min_ (*P* < 0.001) in those with CTEPH. This is explained by the load‐dependency of these measurements and the significant rise in ESP (*P* < 0.001) in those with CTEPH whilst EDP (*P* = 0.162) remained constant.

**Table 3:** Demographics, right heart catheterization and RV hemodynamic data when classifying patients by mPAP at rest

	mPAP ≤ 25 (*n* = 10)	mPAP > 25 (*n* = 10)	*P*‐value
Demographics			
Age, years	55 ± 17	51 ± 14	0.564
Male sex, *N* (%)	4 (40.0)	6 (60.0)	0.656
BMI, kg/m^2^	31.2 ± 8.2	29.2 ± 4.4	0.880
6MWD, m	369 ± 79	377 ± 134	0.902
WHO Class II/III, *N* (%)	10 (100.0)	10 (100.0)	1.000
Right heart catheter			
mPAP, mmHg	18.8 ± 5.5	39.7 ± 7.1	<0.001
Systolic PAP, mmHg	30.7 ± 9.6	66.8 ± 16.4	<0.001
Diastolic PAP, mmHg	10.1 ± 4.8	24.1 ± 6.6	<0.001
mRAP, mmHg	5.8 ± 4.2	8.0 ± 3.5	0.109
PCWP, mmHg	8.3 ± 4.3	10.8 ± 4.9	0.244
RV EDP, mmHg	5.8 ± 3.5	10.1 ± 3.4	0.012
SvO_2_, %	74.1 ± 4.2	71.5 ± 6.3	0.305
CO, L/min	5.3 ± 1.3	4.7 ± 1.0	0.249
CI, L/min/m^2^	2.6 ± 0.3	2.4 ± 0.6	0.334
PVR, dyne/sec/cm^5^	163 ± 57	573 ± 220	<0.001
RV Hemodynamics			
Heart Rate, BPM	68 ± 10	71 ± 13	0.560
SW, mmHg.mL	1402 ± 500	2018 ± 732	0.041
CO, L/min	5.3 ± 1.4	4.9 ± 1.0	0.436
ESP, mmHg	34.2 ± 7.1	67.4 ± 16.6	<0.001
EDP, mmHg	10.3 ± 4.3	13.1 ± 4.4	0.162
ESV, mL	111.0 ± 44.4	85.2 ± 26.2	0.241
EDV, mL	149.8 ± 44.4	118.2 ± 37.7	0.104
SV, mL	78.7 ± 22.0	71.8 ± 26.3	0.532
EF, %	51.6 ± 8.3	55.5 ± 9.9	0.354
dP/dt_max_, mmHg/sec	351 ± 76	502 ± 181	0.016
dP/dt_min_, mmHg/sec	‐326 ± 92	‐618 ± 173	<0.001
Ea, mmHg/mL	0.47 ± 0.18	1.10 ± 0.52	0.005
Tau, msec	63.9 ± 17.3	64.3 ± 16.4	0.954
Ees/Ea	2.29 ± 1.68	0.72 ± 0.59	0.02

Values are mean ± SD or *n* (%).

BMI, body mass index; 6MWD, 6 min walking distance; WHO Class, world health organisation classification; mPAP, mean pulmonary arterial pressure; mRAP, mean right atrial pressure; PCWP, pulmonary capillary wedge pressure; RV EDP, right ventricular end‐diastolic pressure; SvO_2_, mixed venous oxygen saturations; CO, cardiac output; CI, cardiac index; PVR, pulmonary vascular resistance; SW, stroke work; ESP, end‐systolic pressure; EDP, end‐diastolic pressure; ESV, end‐systolic volume; EDV, end‐diastolic volume; SV, stroke volume; EF, ejection fraction; dP/dt_max_, maximum rate of isovolumic contraction; dP/dt_min_, maximum rate of isovolumic relaxation; Ea, effective arterial Elastance; Tau, time constant of diastolic relaxation; Ees/Ea, Single‐beat ventriculo‐arterial coupling ratio.

After reclassifying patients according to the Ees/Ea_max sw_ threshold established in the animal model (Table 4), we demonstrated that mPAP (*P* = 0.006) and PVR (*P* = 0.003) were normal in the Ees/Ea ≥ 0.68 group and elevated in the Ees/Ea < 0.68 group. There was a significant increase in HR (*P* = 0.04) in the Ees/Ea < 0.68 group. While Tau (*P* = 0.418) remained constant, there was a significant increase in dP/dt_min_ (*P* = 0.003), again explained by the load‐dependency of this measurement and the significant rise in ESP (*P* < 0.001) whilst EDP (*P* = 0.103) remained constant. Although SW (*P* = 0.853) remained constant between the Ees/Ea subgroups, there was a significant reduction in CO (*P* = 0.024), SV (*P* = 0.006), ESV (*P* = 0.034) and EDV (*P* = 0.007) in the Ees/Ea < 0.68 group. The differences in heart rate and volumetric indices were not identified when patients were stratified by pressure.

**Table 4:** Demographics, right heart catheterization and RV hemodynamic data when reclassifying patients with CTED or CTEPH by Ees/Ea_max sw_ determined from an animal model

	Ees/Ea ≥ 0.68 (*n *=* *11)	Ees/Ea < 0.68 (*n *=* *9)	*P*‐value
Demographics			
CTED, *N* (%)	8 (72.7)	2 (27.3)	0.072
CTEPH, *N* (%)	3 (22.2)	7 (77.7)	0.073
Age, years	55 ± 16	50 ± 16	0.516
Male sex, *N* (%)	5 (45.4)	5 (55.6)	0.653
BMI, kg/m^2^	30.9 ± 7.4	29.1 ± 4.9	0.804
6MWD, m	380 ± 81	364 ± 137	0.747
WHO class (II/III)	11 (100.0)	9 (100.0)	1.000
Right heart catheter			
mPAP, mmHg	22.8 ± 8.0	37.1 ± 12.5	0.006
Systolic PAP, mmHg	38.8 ± 17.1	60.9 ± 23.5	0.026
Diastolic PAP, mmHg	12.4 ± 6.0	22.9 ± 9.1	0.006
mRAP, mmHg	7.1 ± 4.7	6.7 ± 3.0	0.817
PCWP, mmHg	10.8 ± 5.8	8.0 ± 2.2	0.144
RV EDP, mmHg	7.6 ± 4.5	8.3 ± 3.5	0.709
SvO_2_, %	74.5 ± 4.7	71.0 ± 6.0	0.120
CO, L/min	5.3 ± 1.3	4.6 ± 1.0	0.217
CI, L/min/m^2^	2.7 ± 0.3	2.3 ± 0.5	0.082
PVR, dyne/sec/cm^5^	181 ± 78	554 ± 250	0.003
RV hemodynamics			
Heart rate, BPM	65 ± 11	75 ± 10	0.04
SW, mmHg.mL	1683 ± 663	1743 ± 755	0.853
CO, L/min	5.6 ± 1.2	4.4 ± 0.8	0.024
ESP, mmHg	36.7 ± 11.6	68.1 ± 16.7	<0.001
EDP, mmHg	10.2 ± 3.8	13.5 ± 4.7	0.103
ESV, mL	114.0 ± 36.3	78.6 ± 31.4	0.034
EDV, mL	156.1 ± 35.5	107.0 ± 37.1	0.007
SV, mL	87.7 ± 22.1	60.1 ± 16.5	0.006
EF, %	53.3 ± 9.8	54.0 ± 8.8	0.875
dP/dt_max_, mmHg/sec	385 ± 114	475 ± 190	0.173
dP/dt_min_, mmHg/sec	‐358 ± 136	‐612 ± 183	0.003
Ea, mmHg/mL	0.42 ± 0.10	1.22 ± 0.43	<0.001
Tau, msec	67.1 ± 13.7	60.8 ± 19.2	0.418
Ees/Ea	2.38 ± 1.49	0.45 ± 0.14	<0.001

Values are mean ± SD or *n* (%).

CTED, chronic thromboembolic disease; CTEPH, chronic thromboembolic pulmonary hypertension; BMI, body mass index; 6MWD, 6 min walking distance; WHO Class, world health organisation classification; mPAP, mean pulmonary arterial pressure; mRAP, mean right atrial pressure; PCWP, pulmonary capillary wedge pressure; RV EDP, right ventricular end‐diastolic pressure; SvO_2_, mixed venous oxygen saturations; CO, cardiac output; CI, cardiac index; PVR, pulmonary vascular resistance; SW, stroke work; ESP, end‐systolic pressure; EDP, end‐diastolic pressure; ESV, end‐systolic volume; EDV, end‐diastolic volume; SV, stroke volume; EF, ejection fraction; dP/dt_max_, maximum rate of isovolumic contraction; dP/dt_min_, maximum rate of isovolumic relaxation; Ea, effective arterial Elastance; Tau, time constant of diastolic relaxation; Ees/Ea, single‐beat ventriculo‐arterial coupling ratio.

**Figure 4:**
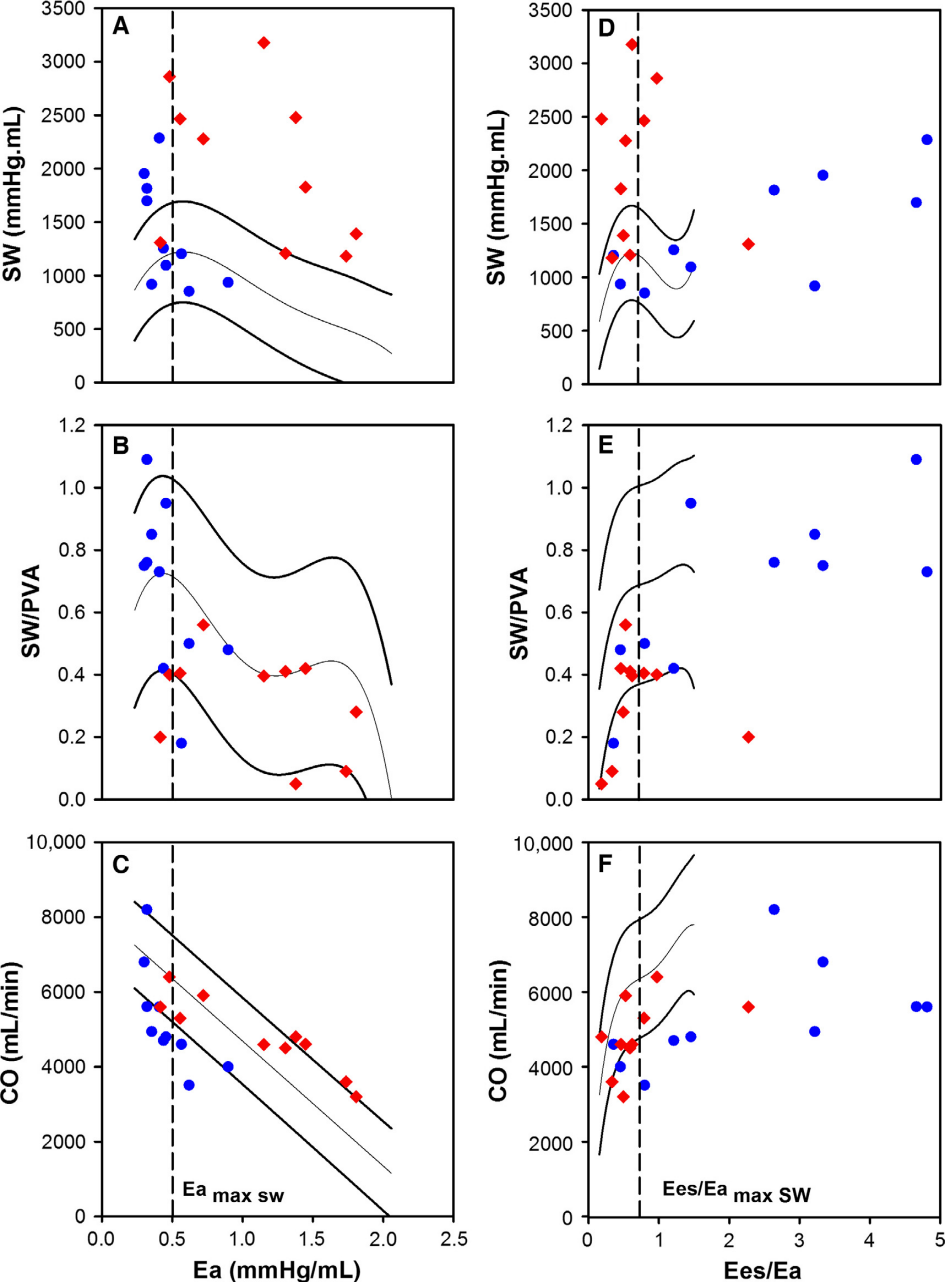
The individual data points for the patients with CTED (*n* = 10) or CTEPH (*n* = 10) overlaid on top of the mean regression lines determined from the animal model for the relation between afterload quantified by Ea and (A) SW; (B) SW/PVA; and (C) CO. The individual data points for the patients with CTED (*n* = 10) or CTEPH (*n* = 10) overlaid on top of the mean regression lines determined from the animal model for the relation between ventriculo‐arterial coupling ratio (Ees/Ea) and (D) SW; (E) SW/PVA; and (F) CO.

Two patients with CTED were identified with an Ees/Ea < 0.68 indicating occult RV pathology (Fig. 4) and three CTEPH patients still had residual RV energetic reserve (Ees/Ea ≥ 0.68). This equated to a reclassification of 25% of the entire cohort. The Ees/Ea coupling ratio for these three misclassified CTED patients was 0.35 and 0.45, even though they presented with normal mPAP (15 and 19 mmHg) and PVR (157 and 230 dyne/sec/cm^5^).

**Figure 5:**
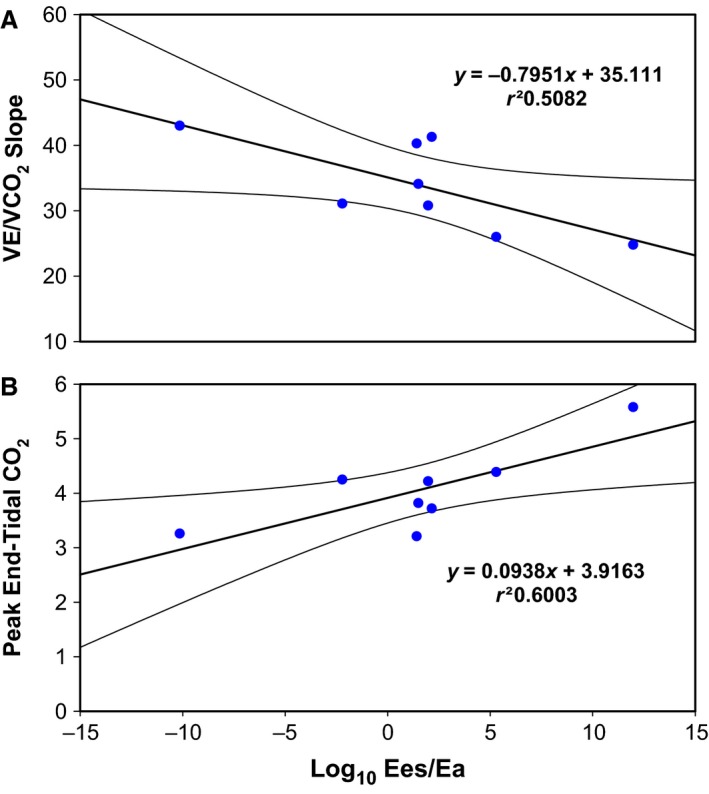
The individual data points for the patients with CTED (*n* = 8) for the relation between Ees/Ea and cardiopulmonary exercise (A) VE/VCO_2_ and (B) peak end‐tidal CO_2_. Linear regression lines with 95% confidence intervals are displayed.

Cardiopulmonary exercise test data for patients with CTED and CTEPH are displayed in Table S1 showing exercise capacity assessed by VO_2_ (%) was marginally higher in patients with CTED. Ees/Ea related to both VE/VCO_2_ slope and peak end‐tidal CO_2_ (Fig. 5) in patients with CTED but showed no relationship to peak VO_2_ (*r* = −0.12, *P* > 0.05).

## Discussion

This is the first study to use the conductance technique to first determine the interaction between the RV and PA in a large animal model to define an Ees/Ea threshold for maximal SW, and then applied it in a cohort of patients with CTED or CTEPH to help re‐characterize RV pathophysiology. The interaction between the RV and the PA determines the external work and metabolic efficiency of the ventricle. Animal model data have shown that the Ees/Ea_max sw_ was 0.68 ± 0.23, beyond which RV dysfunction ensued. Redefining patients with CTED and CTEPH using this Ees/Ea threshold revealed three patients with CTEPH who had an Ees/Ea ≥ 0.68, demonstrating RV energetic reserve and two patients with CTED who had Ees/Ea < 0.68. This suggests occult RV dysfunction that was not apparent on resting right heart catheterization. On exercise, CTED patients with lower Ees/Ea also developed greater exercise ventilatory inefficiency with an increased hyperventilatory response. This supports a possible link between resting RV dysfunction detected by conductance catheterization and unfavorable RV adaptation to exercise. These findings provide novel insight into RV energetics observed in CTED and CTEPH, potentially improving on the current disease definition in CTED and identifying those with RV maladaptation.

### Animal model

We establish that while maximal efficiency is important from the perspective of understanding ideal energy transfer conditions between RV work and oxygen consumption, the threshold of maximal SW is the determinant factor in RV failure. We have demonstrated acute RV adaptation in response to acute changes in afterload. There is an immediate response where the RV systolic pressure rises to maintain ejection. Then, as the afterload continues to increase, there are only relatively small increases to EDP and EDV as the RV dilates and distends to accommodate the increasing systolic pressure required to attempt to facilitate ejection. As the systolic pressure rises beyond the threshold point of maximal SW, ESV increases and combined with the relatively small change in EDV, this results in a reduction in SV. This reduction in SV causes a decline in CO and SW, as the RV fails to maintain sufficient output against the increasing loading conditions. Therefore, in terms of efficiency and SW, as the afterload increases from baseline, RV output is adequately maintained through the arbitrary point of maximal efficiency until the loading conditions reach a threshold point of maximal SW at an Ees/Ea = 0.68. Beyond this threshold of maximal SW, SW and CO may continue to offer sufficient output to meet demand at rest. However, if the afterload loading conditions are increased further (as is observed during modest exercise), there is insufficient RV energetic reserve and the RV will fail to meet demand. Ees/Ea can be used as a surrogate load‐independent indicator for RV failure. Evaluating the Ees/Ea at maximal SW could better determine disease severity and guide management.

We have established a threshold for RV‐PA ventriculoarterial uncoupling in an animal model, beyond which, there is insufficient energetic reserve to maintain adequate RV function. Theoretical models have predicted optimal mechanical coupling corresponded to an Ees/Ea = 1 (Sunagawa et al. 1984); with current literature suggesting in an healthy individual RV‐PA ventriculoarterial coupling is between 1 and 2 (Naeije and Manes 2014) and that the values for the LV and RV are comparable. While we demonstrate agreement with previous work at baseline (Guihaire et al. 2013), with slight uncoupling between the RV‐PA at rest (Ees/Ea = 0.94 ± 0.18), our work has shown that maximal SW occurs at an Ees/Ea = 0.68 ± 0.23, and that uncoupling only occurred beyond this threshold as there was insufficient RV energetic reserve to maintain function as Ea increased further. This finding is supported by previous work (Fourie et al. 1992) where authors suggested the RV uncoupled around an Ees/Ea = 0.6 mmHg/mL. One explanation for this apparent inefficient energy transfer between the RV and PA could be the complex pulmonary vascular structure. Whereas, the LV is coupled with an elastic aorta and all energy expended during the pulsatile contraction is converted into flow energy due to the elastic recoil of the aorta (Yaginuma et al. 1985; Sasayama and Asanoi 1991), the pulmonary artery has relatively short elastic proximal vessels which branch into the pulmonary trunk, so that 25–40% of the energy expended during the contraction of the RV is not converted into flow energy due to the restricted elastic recoil of the pulmonary vessels (Grignola et al. 2007; Saouti et al. 2010). This would explain the lower coupling ratio we observed.

### Translation into the clinical cohort

CTED is poorly defined and as a result is difficult to diagnose and treat. This study for the first time evaluates not only Ees/Ea but also RV efficiency (SW/PVA) in this condition. We used an animal model to define the Ees/Ea coupling ratio at maximal SW and, in one analysis, apply this threshold to re‐evaluate patients with CTED and CTEPH by assessing RV function. Previous studies have relied on single‐beat estimation methodologies to determine Ees from a real end‐systolic point on the baseline PV‐loop and a second theoretical point (Bellofiore and Chesler 2013). This enables RV‐PA ventriculoarterial‐coupling ratio adaptation in response to increased loading conditions in PH to be observed. Our group has used this methodology to confirm that ventriculoarterial‐coupling is maintained in patients with CTED. Proportional increases in both Ees and Ea occur in CTED, whereas in patients with CTEPH, Ea increases disproportionately and the RV becomes uncoupled from the PA (McCabe et al. 2014). The inability to adapt and increase resting contractility (Ees) in the presence of increased afterload also appears to be a particular problem in patients with systemic sclerosis and PH resulting in greater uncoupling in this cohort as well (Tedford et al. 2013).

Applying an Ees/Ea coupling ratio threshold derived from our animal model suggested that in the majority of our CTED patients, the RV retained its energetic reserve whereas in only two patients, resting RV function appears to be compromised. Conversely whilst most patients with CTEPH have significant RV dysfunction, a few retain a RV energetic reserve. After reclassification, those patients with Ees/Ea uncoupling suffer a reduced SV, and therefore, HR and ESP is significantly increased to maintain adequate CO at rest. Reclassification was made in 25% of our cohort which may be clinically important. In those CTED patients that lack RV energetic reserve, endarterectomy or pulmonary vasodilator therapy may be indicated.

An implication of reduced RV energetic reserve in CTED may be an impaired RV exercise response, giving rise to impaired exercise ventilation‐perfusion matching. This is supported by higher VE/VCO_2_ slope gradients in patients with low Ees/Ea values. Higher end‐tidal CO_2_ values at anaerobic threshold also suggest preserved cardio‐ventilatory coupling with a lower hyperventilatory response to exercise in patients with preserved Ees/Ea values. A recent study of 42 patients with CTED undergoing pulmonary endarterectomy demonstrated an improvement in 6‐min walk distance of 49 m at 12 months post‐operatively (Taboada et al. 2014). This effect may have been exaggerated with more accurate triaging by assessing V‐A coupling. This study alludes to dynamic interplay between the constituents of pulmonary afterload and exercise capacity in CTED as post‐operative changes in routine assessed pulmonary hemodynamics were relatively small. Further work studying RV energetics and treatment response is therefore justified in a larger population of CTED patients.

### Study limitations

Some limitations of our study must be recognized. The porcine model of RV‐PA ventriculoarterial interaction has an open pericardium that reduces RV contractility and partly explains why porcine PVR is higher than in humans. However, this remains the best physiological model of assessing the response of normal RV function to changes in afterload, as LV‐RV interdependence is retained. Furthermore, fentanyl can adversely affect contractility and we inserted the conductance catheter apically in the animal model that may have further diminished RV contractility. Nevertheless, it remains the most commonly studied model (Kuehne et al. 2004; Grignola et al. 2007; Read et al. 2011; Simonneau et al. 2013; McCabe et al. 2014). The animal model assessed the effect of acute afterload perturbation on RV function whereas in the human model the RV is chronically loaded and the influence of remodelling in the human model is a possible and important unaccounted confounder. We were not able to insert an additional venous sheath for IVC balloon occlusion in the clinical studies. Therefore, we used single beat methodology to determine the Ees/Ea coupling ratio. While this method has not been validated in the RV of patients with PH, it remains commonly used by others (Kuehne et al. 2004; Herberg et al. 2013; McCabe et al. 2014). This small pilot study will need to be repeated in a larger clinical trial to fully determine the clinical implications of our findings and in particular to better understand RV and gas exchange exercise characteristics in CTED. We were unable to determine the prognostic significance of using Ea as a marker of RV dysfunction in this and other causes of PH and this remains the focus of further research. Finally, patients did not have biventricular conductance measurements performed and we cannot comment on the effect of ventricular interdependence on our findings. All the patients studied had confirmed normal LV function on echocardiography and normal capillary wedge pressures at right heart catheterization. It is unlikely that LV dysfunction unduly influenced the functional changes observed in the RV.

## Conclusions

The Ees/Ea coupling ratio at maximal SW determined from an animal model defined an Ees/Ea threshold of 0.68, beyond which RV dysfunction occurs. Using this threshold we have detected occult RV pathology at rest in patients with otherwise normal pulmonary hemodynamics, and demonstrated RV energetic reserve in some cases of CTEPH. Detailed Ees/Ea coupling data may improve the diagnostic accuracy of patients with chronic pulmonary thromboemboli and usefully influence their management.

## Conflict of Interest

None. There are no relationships with industry.

## Data Accessibility

## Supporting information




**Table S1**. Cardiopulmonary exercise testing data when classifying patients by mPAP at rest.
