# Supplementary material for: Ventriculo‐arterial coupling detects occult RV dysfunction in chronic thromboembolic pulmonary vascular disease
Source: Physiol Rep. 2017 Apr 3;5(7):e13227. doi: 10.14814/phy2.13227 (PMC5392517; doi:10.14814/phy2.13227)
Supplement: Supplementary file 1 — Table S1. Cardiopulmonary exercise testing data when classifying patients by mPAP at rest. [file PHY2-5-e13227-s001.docx]

**Table S1.** Cardiopulmonary exercise testing data when classifying patients by mPAP at rest.

|  |  | CTED (mPAP < 25mmHg) (n = 8) | CTEPH (mPAP > 25mmHg) (n = 8) | P value |
| --- | --- | --- | --- | --- |
|  | VO_2_, ml/Kg/min | 19.2 ± 4.8 | 16.1 ± 4.7 | 0.390 |
|  | VO2, % | 72 ± 16 | 88 ± 12 | **0.047** |
|  | HR, beats/min | 146 ± 22 | 154 ± 12 | 0.341 |
|  | VE, l/min | 72 ± 21 | 84 ± 30 | 0.367 |
|  | RER | 1.17 ± 0.10 | 1.12 ± 0.12 | 0.386 |
|  | O_2_ pulse, ml/beat | 10.7 ± 3.4 | 8.9 ± 3.0 | 0.278 |
|  | VE/VCO_2_ slope | 33.9 ± 7.0 | 48.2 ± 13.6 | **0.020** |
|  | etCO_2,_ (AT) kPa | 4.4 ± 0.6 | 3.4 ± 0.5 | **0.004** |

Values are mean ± S.D.

VO_2_ – oxygen uptake; HR – heart rate; VE – minute ventilation; RER – respiratory exchange ratio; O2 pulse – predicted VO_2 max_/ predicted maximum HR; VE/VCO_2_ – minute ventilation/carbon-dioxide production; etCO_2_ (AT) – end-tidal carbon-dioxide at anaerobic threshold
